# Organoids Gone Viral: A Comprehensive Review on Human Organoid Models to Study Viral Pathogenesis

**DOI:** 10.3390/v18020238

**Published:** 2026-02-13

**Authors:** N. S. Suneesh, Parikshit Bagchi, Anupam Mukherjee

**Affiliations:** 1 ICMR-National Institute of Virology, Pune 411001, India; suneesh.nari24a@acsir.res.in; 2 AcSIR-Academy of Scientific & Innovative Research, Ghaziabad 201002, India; 3 Department of Molecular Microbiology, Washington University School of Medicine in St. Louis, St. Louis, MO 63110, USA

**Keywords:** organoids, viral pathogenesis, host–virus interactions, human stem cell-derived models, emerging and re-emerging viruses, antiviral drug discovery, translational virology, 3d cell culture models

## Abstract

Organoid technology has transformed experimental virology by offering physiologically relevant 3D human models that bridge the gap between conventional 2D cell cultures and complex in vivo systems. Derived from pluripotent or adult stem cells, organoids self-organize into multicellular structures that recapitulate native tissue architecture and function, enabling more accurate modeling of host–virus interactions and disease mechanisms. This review outlines the evolution and application of organoid-based systems across neural, intestinal, hepatic, pulmonary, and renal tissues for studying a broad range of human viruses that remain a public health burden. These models can reproduce viral tropism, immune signaling, and host variability, offering new molecular insights into infection dynamics. Integration with single-cell transcriptomics, CRISPR editing, and antiviral screening has expanded the translational utility of organoids, establishing them as a powerful platform for antiviral discovery, vaccine testing, and precision medicine.

## 1. Introduction

Viral diseases have been plaguing humankind for thousands of years, and their frequency and impact have grown exponentially in recent decades owing to factors such as rapid evolution of viruses, ecological disturbances, rapid urbanization, and human migration [1,2,3]. In addition to these factors, the effects of climate change are becoming increasingly obvious with every passing year, especially as it disrupts the ecological system and blurs the lines separating species. Projections show that by 2070, due to the drastic shift in global temperatures, thousands of species will converge on biodiversity hotspots and regions of dense human population, which exponentially increase the chances of cross-species viral transmission and the risk of pandemic [4,5,6]. In these recent years alone, we have witnessed the resurgence of viral threats like Zika, Ebola, MERS-CoV, Dengue, and Chikungunya [7,8]. Simultaneously, novel viral entities, such as the Severe Fever with Thrombocytopenia Syndrome virus (SFTS), have raised concern for humans [9]. These changing dynamics in viral evolution and the increasing susceptibility of the human population underscore an urgent need for scalable, physiologically sound models to study host–virus interactions.

Understanding how these emerging and re-emerging viruses transmit and cause specific diseases has been the primary focus of biomedical research in recent years, especially following the SARS-CoV-2 pandemic in 2019 [10,11]. Understanding viral transmission patterns and the molecular mechanisms driving pathogenesis is critical for predicting outbreaks and developing a vaccine. Despite significant advances, effective antiviral options remain limited for many viruses, including oncogenic pathogens, where persistent infection and tissue-specific disease progression remain poorly modeled in conventional systems, underscoring the need for adjunct and host-directed therapeutic strategies alongside conventional approaches [12,13,14,15,16]. Current virology models, such as immortalized cell lines and animal systems, are constrained by their inability to accurately mimic human physiological conditions, including tissue architecture, cellular heterogeneity, and authentic viral replication dynamics [17,18]. Consequently, there has been a growing demand for improved human-relevant experimental platforms.

In recent years, organoids have emerged as a promising solution to this challenge. Organoids provide powerful platforms for studying viral infections in settings that closely resemble human physiology [19]. These models have been successfully applied to investigate many human viruses, yielding critical molecular insights into viral replication and host responses that were previously inaccessible [20,21]. Organoids are rapidly becoming an indispensable tool in virology, enabling more accurate disease modeling, improved antiviral testing, and potential applications in personalized medicine that were scarcely conceivable a decade ago.

## 2. What Are Organoids?

An organoid is a three-dimensional (3D), self-organized tissue construct generated *in vitro* from stem cells, which may be pluripotent, fetal, or adult in origin, and that captures the key cytoarchitectural, functional, and biological features defining an organ [22,23,24]. Key hallmarks of an organoid include their ability to organize into complex tissue architecture, differentiate, and mimic the properties of a typical organ, making them a middle ground between 2D cell cultures and relevant in vivo systems [25,26,27,28]. The cells used for organoid generation can be derived from various sources, such as induced pluripotent stem cells (iPSCs) or tissue-derived cells (TDCs), including normal stem or progenitor cells, differentiated cells, or even cancerous cells [29,30,31]. Organoids are broadly classified into two origins: pluripotent stem cells (PSCs), including ESCs, which offer high developmental flexibility and recapitulate early organogenesis, and adult stem cells (ASCs), which generate tissues restricted to specific lineages [32,33,34,35]. However, PSC-derived organoids typically require longer culture periods, and the resulting structures often resemble early fetal developmental stages [34,36,37,38].

Early studies showed that dissociated cells can self-organize, beginning with Wilson’s 1907 sponge experiments and Barth and Holtfreter’s findings that embryonic ectodermal cells could reaggregate and differentiate in simple culture conditions [39,40,41]. By the late 20th century, key stem-cell breakthroughs, most notably the derivation of mouse ESCs (1981) and human ESCs (1998), showed that pluripotent cells could be stably maintained *in vitro* with full self-renewal and differentiation potential [42,43]. About 25 years later, Takahashi and Yamanaka (2006) showed that introducing four factors-Oct3/4, Sox2, c-Myc, and Klf4-could reprogram fibroblasts into induced pluripotent stem cells (iPSCs), opening a new era in stem cell biology [44]. Building on these advances, Eiraku et al. (2008) showed that mouse and human ESCs grown in 3D suspension could self-organize into polarized cortical tissues that recapitulate early corticogenesis [45].

**Figure 1:**
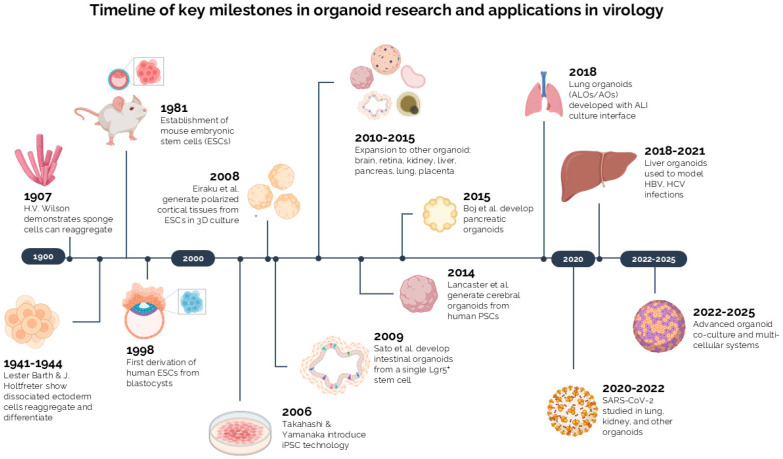
Timeline of Organoid Development and Applications in Virology. Chronological overview of key milestones in organoid research, from early demonstrations of cellular self-organization (1907–1944) to the advent of stem cell and iPSC technologies (1981–2006), establishment of pluripotent and adult stem cell-derived organoids (2008–2009), and their expansion to multiple organs (2010–2015) [31,39,41,44,45,47,50]. The timeline also highlights the integration of organoid systems into virology, enabling studies of viral pathogenesis, host–virus interactions, and antiviral testing (2018–2025).

The modern era of organoid research began in 2009 with the landmark study by Sato et al., which demonstrated that a single Lgr5^+^ intestinal stem cell could generate a complete crypt-villus architecture *in vitro* [31]. These “mini-guts” exhibited long-term self-renewal, formed functional crypt-villus structures, and did not require a mesenchymal niche, establishing the first robust adult epithelial organoid system. This breakthrough, driven by Wnt activation and BMP inhibition, catalyzed the development of organoid models across multiple organ systems and firmly positioned them as physiologically relevant alternatives to traditional 2D cultures [46,47,48,49,50,51,52,53]. The evolutionary timeline of organoid technology, from foundational discoveries to its application in virology, is schematically presented in Figure 1.

## 3. Organoids in Virology

The application of organoid technology in virology has followed a trajectory distinct from its original use in developmental biology and cancer research. While early organoid studies primarily focused on cell fate specification, tissue morphogenesis, and oncogenic transformation, virologists rapidly recognized the value of these systems as physiologically relevant human models for studying host–virus interactions and viral pathogenesis.

Organoid models have enabled detailed interrogation of viral replication cycles, cell-type-specific tropism, immune evasion strategies, and tissue-level responses that are not achievable with conventional 2D cell cultures or many animal models. By preserving key features of native tissue architecture, cellular heterogeneity, and epithelial polarity, organoids provide experimental contexts that more accurately reflect human infection dynamics. As a result, organoid-based systems have become indispensable tools for investigating viral pathogenesis, congenital infection, and antiviral responses in human-relevant tissues where traditional models have proven insufficient. The following sections review organoid-based virology studies across distinct organ systems, organized by tissue context and viral tropism, and summarize key experimental insights enabled by these platforms.

### 3.1. Brain Organoids for Neurotropic Viruses

Brain organoids are three-dimensional (3D), self-organizing structures generated from pluripotent stem cells that recapitulate key features of human brain development, including tissue architecture, regional patterning, and neuronal differentiation [18,54].

Neurotropic viruses preferentially infect neurons and glial cells, thereby targeting both the central and peripheral nervous systems. Infection of these cell types can initiate a range of neurological pathologies, in some cases leading to severe or fatal outcomes [55,56]. Studying neurotropic viruses remains challenging due to several constraints, such as limited access to developing brain tissue, virus-specific cellular tropism, and the inability of conventional 2D cultures or animal models to capture the complexity of human neurodevelopment and neural cytoarchitecture [57,58,59].

#### 3.1.1. Zika Virus (ZIKV)

Zika virus (ZIKV) is an enveloped, positive-sense single-stranded RNA (+ssRNA) virus belonging to the family *Flaviviridae*. ZIKV is highly neurotropic, and its replication cycle involves direct infection of neural progenitor cells, astrocytes, and radial glial cells [60,61,62,63,64]. The ZIKV outbreak of 2015–2016 was a turning point in establishing organoids as an essential tool for developing virology models, especially for congenital microcephaly. ZIKV infection of brain organoids modeling early fetal brain development preferentially targeted neural progenitor cells (NPCs), leading to cell death, neurogenesis impairment, growth suppression, and reduced organoid size, closely recapitulating microcephaly associated phenotypes [65,66,67]. This disruption of neurogenesis was associated with Toll-like Receptor 3 (TLR3) signaling [65]. Forebrain-specific organoids developed using a miniaturized bioreactor system demonstrate that ZIKV preferentially infects neural progenitor populations, thereby impairing their proliferation and survival [68]. The neuroprotective effect of Betulinic Acid was shown to suppress ZIKV-induced cell death by activating the Akt signaling pathway in NPCs and organoids [69]. ZIKV also demonstrated unexpected therapeutic potential, exhibiting oncolytic activity in Glioblastoma (GBM) stem cells via the SOX2-integrin α_v_β_5_ axis [70]. However, the mechanism of viral entry remains unknown, as gene knockout of the previously reported receptor Axl did not protect NPCs and brain organoids from ZIKV infection [71].

#### 3.1.2. Herpes Simplex Virus Type-1

Herpes Simplex Virus type 1 (HSV-1) is a double-stranded DNA virus belonging to the family *Orthoherpesviridae* and exhibits marked neurotropism. Following primary infection and lytic cycle in mucosal epithelial cells, HSV-1 can establish latency in the sensory neurons of the trigeminal ganglia [72,73,74,75]. Using hiPSC-derived 2D neuronal cultures and brain organoids, latent HSV-1 infection has been modeled in systems that recapitulate in vivo tissue architecture and exhibit restricted viral reactivation. In parallel, separate studies demonstrated that HSV-1 infection of brain organoids induces pathological features resembling neurodevelopmental disorders, including impaired neuronal differentiation, disrupted cortical layering, aberrant microglial activation, and inflammation [76,77]. HSV-1 infection was associated with the accumulation of Aβ42, a hallmark of Alzheimer’s disease (AD), in hiPSC-derived brain organoids, whereas this phenotype was not observed in 2D cultures [78]. In another line of approach, brain organoids developed by seeding human-induced neural stem cells (hiNSCs) into a silk porous scaffold and infected with HSV-1 similarly exhibited Alzheimer’s disease-like features, including Aβ accumulation, upregulation of PSEN1/PSEN2, reactive gliosis, neuronal loss, and neuroinflammation, consistent with findings observed in hESC-derived HSV-1-infected brain organoids [74]. Further, HSV-1 infection has been found to disrupt neuroepithelial integrity and to evade type I interferon responses via ICP34.5-mediated mechanisms, leading to altered transcriptional patterns [79].

Another study using hiPSC-derived organoids and single-cell RNA sequencing to understand HSV-1 infection dynamics showed significant enrichment of TNF signaling, which was suppressed following treatment with acyclovir in combination with necrostatin-1 or bardoxolone methyl (CDDO-Me), correlating with attenuation of HSV-1-associated neuropathological features [80]. The CRISPR-Cas9 tool was employed to target ICP0 or ICP27, resulting in a significant reduction in latent infection in brain organoids [81].

#### 3.1.3. Severe Acute Respiratory Syndrome-Corona Virus-2 (SARS-CoV-2)

Severe acute respiratory syndrome coronavirus 2 (SARS-CoV-2) is a +ssRNA virus belonging to the family *Coronaviridae* and was the causative agent of the COVID-19 pandemic [82,83]. Although SARS-CoV-2 primarily targets the respiratory system, it has also been implicated in neurological manifestations, including stroke, anosmia, encephalopathy, and cognitive impairment [84,85,86]. Experimental studies using iPSC-derived brain organoids demonstrated that SARS-CoV-2 can infect neuronal cells and reduce neural progenitor cell (NPC) proliferation in neurospheres, with infection occurring predominantly in cortical neural cells expressing TUJ-1 and NESTIN^+^ NPCs [87]. Supporting these findings, another study reported preferential infection of more mature cortical neurons expressing TUJ1, tau, MAP2, and S100β, with limited involvement of Iba-1^+^ microglia, indicative of organoid maturation; neurodegeneration was evident within 2 days post-infection (dpi) [88]. Investigations using choroid plexus organoids (CPOs) that express ACE2 and TMPRSS2 revealed heightened susceptibility to SARS-CoV-2, accompanied by pronounced cytopathic effects (CPE) [89]. Complementary studies employing choroid plexus-CSF organoids that secrete cerebrospinal fluid confirmed similar vulnerability, collectively highlighting the choroid plexus as a critical site of SARS-CoV-2-associated neuropathology [90].

Further advances in cortical organoid systems incorporating pericyte-like cells (PLCs) and endothelial components identified vascular structures as sites supporting viral replication, with subsequent viral spread to astrocytes and activation of type I interferon signaling. Mechanistic analyses revealed that SARS-CoV-2 entry into astrocytes can occur via ACE2-independent pathways, involving neuropilin-1 (NRP1) and two-pore segment channel 1 (TPCN1), potentially explaining the variable viral tropism observed across organoid models at different maturation stages [91,92]. Altered astrocyte responses were also observed, including disruption of neurotransmitter homeostasis, metabolic stress, and altered transcriptional dynamics associated with astrogliosis, inflammation, and cell survival [93,94]. A study investigating SARS-CoV-2 variants of concern (VOCs) using organoid models demonstrated that viral tropism evolved with genetic changes; Omicron sublineages BA.2 and BA.5 exhibited enhanced replication efficiency and increased cytotoxicity compared with earlier strains, including the Delta variant, underscoring the role of viral evolution in neuropathology [95,96]. Beyond mechanistic investigations, brain organoids have also been used for antiviral evaluation, in which treatment with remdesivir and sofosbuvir suppressed viral replication and preserved neural cell viability [97].

#### 3.1.4. Human Cytomegalovirus (HCMV)

Human Cytomegalovirus (HCMV) is a dsDNA virus belonging to the family *Orthoherpesviridae* and causes congenital birth defects, such as sensorineural hearing loss and microcephaly [98,99]. The advent of brain organoid platforms enabled detailed investigation of HCMV-induced microcephaly, revealing that, similar to other neurotropic viruses, HCMV infection results in severe disruption of cortical architecture and neural development. Infection of brain organoids led to marked loss of structural integrity, necrosis, vacuolation, and atypical neuronal differentiation, hallmarks of congenital brain dysfunction [100,101]. Subsequent studies using iPSC-derived brain organoids identified TBR2^+^ neural progenitor cells (NPCs) within the subventricular zone as primary viral targets, linking their infection to impaired organoid growth and aberrant neural activity [102]. Transcriptomic analyses in hiPSC-derived brain organoids further demonstrated that HCMV infection downregulates key transcription factors, including FEZF2, EMX1, and FOXG1, thereby disrupting telencephalic and cortical fate specification, even in cells exhibiting minimal viral protein expression. These findings suggest that widespread developmental dysregulation may be driven by viral proteins or paracrine signaling, independent of productive viral replication [103]. Additionally, studies using 3D cortical organoids showed that mitochondrial respiration-mediated disruption of tissue architecture and neuronal differentiation is exacerbated by nitric oxide, despite its antiviral properties [104]. Similarly, iPSC-derived cerebral organoids revealed that HCMV-associated downregulation of Nidogen-1 (NID1), a basement membrane protein essential for neuronal tube-like structure formation, contributes to early rosette abnormalities. These findings indicate that viral modulation of host scaffolding proteins plays a critical role in severe neurodevelopmental defects [105,106]. Beyond developmental pathology, HCMV has also been implicated in cancer progression and neurodegeneration using cancer tissue-derived glioblastoma organoids (GBOs), where EphA2 was identified as a viral entry receptor [107]. A potential link between Alzheimer’s disease and HCMV infection was reported in 2024 using brain organoid models, demonstrating that amyloid and tau pathology were driven by CD83^+^ microglia associated with HCMV and IgG4 signatures [108]. Collectively, these organoid-based studies establish HCMV as a potent regulator of neurodevelopmental and neurodegenerative processes, influencing cortical architecture, signaling networks, progenitor dynamics, and even adult neural homeostasis.

#### 3.1.5. Dengue Virus (DENV)

Neuropathogenesis of DENV was investigated using co-cultures of iPSC-derived brain organoids and microglia, revealing that microglia play a critical role in mounting inflammatory responses, with elevated expression of IL-6, IL-1β, TNF-α, and CCL2 [109]. In parallel, the antiviral potential of the oxoaporphine alkaloid hernandonine against DENV-2 was demonstrated, showing that it targets cholesterol-rich lipid rafts during early stages of infections [110].

#### 3.1.6. Japanese Encephalitis Virus (JEV)

Japanese Encephalitis Virus (JEV) is a positive-sense single-stranded RNA (+ssRNA) virus belonging to the family *Flaviviridae* and causes central nervous system infection [111]. JEV infection was established in hESC-derived cortical organoids, demonstrating that NPC-rich organoids younger than four weeks exhibited impaired interferon signaling and heightened susceptibility in a stage-dependent manner, whereas organoids older than eight weeks mounted RIG-I, IFN-β, and STAT1 responses. Developmental impairment was characterized by preferential infection of outer radial glial cells and induction of a microcephaly like phenotype [112].

#### 3.1.7. Measles Virus (MeV)

Measles Virus (MeV) is a negative-sense single-stranded RNA (−ssRNA) virus belonging to the family *Paramyxoviridae* and causes severe pneumonia and encephalitis [113,114]. Hyperfusogenic MeV variants carrying mutations in the fusion (F) protein were investigated using brain organoids. Specifically, the L454W substitution promoted receptor-independent cell fusion and enhanced CNS propagation, recapitulating fatal measles inclusion body encephalitis (MIBE). This mutation was identified as a key molecular determinant of MeV neurotropism and antiviral resistance [115].

#### 3.1.8. La Crosse Virus (LACV)

La Crosse Virus (LACV), a negative-sense RNA *orthobunyavirus* associated with pediatric encephalitis, has also been modeled using brain organoids [116,117]. LACV-infected organoids exhibited pronounced apoptosis in differentiated neurons, which correlated with reduced type I interferon responses compared with neural progenitor cells; neuronal viability was restored following treatment with recombinant interferon. In a complementary study using iPSC-derived brain organoids, a compound was identified that inhibited viral replication by blocking virion trafficking, demonstrating the utility of brain organoids for antiviral drug discovery [118,119].

#### 3.1.9. JC Polyomavirus (JCpyV)

JC Polyomavirus (JCpyV), the causative agent of progressive multifocal leukoencephalopathy (PML) in immunocompromised individuals, has also been investigated using brain organoids [120,121]. Productive infection was achieved by inoculating iPSC-derived 3D brain organoids containing neurons, astrocytes, and oligodendrocytes with the MAD4 strain of JCPyV, resulting in PML-like pathological features [122].

Across neurotropic viruses, brain organoid studies consistently reveal convergence on neural progenitor vulnerability, disruption of cortical organization, and dysregulated inflammatory signaling, while also highlighting virus-specific differences in cell-type tropism, immune evasion strategies, and long-term neuropathological outcomes. These findings underscore both shared and distinct mechanisms of viral neurotropism. Crucially, brain organoids have succeeded where conventional 2D cultures fall short by recapitulating complex neurodevelopmental phenotypes. For instance, although 2D systems can support viral replication, they cannot model the structural ‘microcephaly-like’ reduction in tissue size observed in ZIKV infection or the complex spatial accumulation of amyloid-beta (Aβ42) plaques characteristic of HSV-1 infection. Collectively, these findings demonstrate that the 3D cytoarchitecture of organoids is essential for uncovering virus-induced neuropathological mechanisms. A summary of neurotropic virus studies using brain organoids is provided in Table 1.

### 3.2. Intestinal Organoids for the Study of Enteric Viruses

Intestinal organoids, also known as enteroids or mini-guts, are 3D epithelial structures generated either from Lgr5^+^ adult stem cells or differentiated from human pluripotent stem cells [31,124]. These systems accurately mimic the intestinal epithelium, forming crypt-villus domains that comprise all major cell types, including enterocytes, goblet cells, Paneth cells, and enteroendocrine cells [125]. Their development marked a major advance in enteric virology, especially for previously “unculturable” pathogens like human norovirus (HuNoV), which could not be propagated in transformed cell lines [126]. These intestinal organoids preserve native apical-basolateral polarity, enabling physiologically relevant studies of viral entry, egress, and directional spread [127]. Studies using these models have identified key host determinants of infection, such as histo-blood group antigens and bile acids, that influence viral attachment and replication [128]. In addition, intestinal organoids permit detailed analysis of epithelial-specific innate immune responses, including interferon-mediated restriction of viral replication [129]. By overcoming long-standing culture barriers for major enteric viruses, intestinal organoids have become essential tools for dissecting viral pathogenesis and evaluating antiviral therapeutics [130].

#### 3.2.1. Human Norovirus

Human Norovirus (HuNoV), a positive-sense single-stranded RNA (+ssRNA) enteric virus belonging to the family of *Caliciviridae*, was notoriously difficult to culture using conventional cell culture systems until the introduction of organoid models, particularly enteroids [131]. The development of Human Intestinal Organoids (HIOs: PSC-derived and containing both epithelial and mesenchymal components) and Human Intestinal Enteroids (HIEs: tissue-derived and composed exclusively of epithelial cells) enabled successful HuNoV replication and provided a platform for antiviral testing [130,132]. Early organoid-based HuNoV studies in 2017 demonstrated that PSC-derived HIOs expressed relevant intestinal glycans and bound virus-like particles (VLPs) in a histo-blood group antigen (HBGA)-dependent manner, establishing a physiologically relevant *in vitro* model [133]. Building on this, tissue-derived HIEs differentiated into a 2D monolayer supported consistent viral replication across multiple HuNoV genotypes isolated from clinical samples [132,134]. For certain genotypes, such as GII.3, replication required specific culture conditions, including donor origin, intestinal segment, differentiation state, and bile acid supplementation [135].

Subsequent studies using HIEs revealed that mature enterocytes are the primary targets of HuNoV infection, rather than progenitor cells, and that susceptibility is strongly dependent on secretor status. Enteroids expressing fucosyltransferase 2 (FUT2^+^) were permissive to HuNoV infection, whereas FUT2^−^ enteroids were resistant; CRISPR-mediated knock-in of FUT2 restored susceptibility [128,132,136]. Mechanistic investigations further demonstrated that innate immune responses, particularly type III interferons and downstream interferon-stimulated genes (ISGs), play a central role in restricting viral replication. Disruption of JAK/STAT or MAVS/STAT1 signaling pathways increased HIE susceptibility, whereas interferon treatment suppressed replication in a genotype-dependent manner, with GII.4 strains exhibiting reduced sensitivity compared with GI and GII.3 genotypes [137,138,139]. Hydrophobic bile acids, such as glycochenodeoxycholic acid (GDCA), were identified as critical cofactors enhancing viral attachment and entry via sphingosine-1-phosphate receptor 2 (S1PR2) in the GII.3 genotype. Inhibition of S1PR2 correspondingly inhibited the viral replication in enteroids [135]. These advances have facilitated the application of molecular assays, including qRT-PCR to detect replicative intermediates, and the generation of stable HIE lines for reproducible interrogation of host determinants such as FUT2, STAT1, and MAVS [140,141]. Beyond mechanistic studies, these organoid systems have enabled preclinical antiviral evaluation. Nitazoxanide and its active metabolite tizoxanide suppressed HuNoV replication by activating IRF-1 and inducing ISGs, and acted synergistically with ribavirin, while BTP2, a CRAC channel inhibitor, reduced HuNoV and Tulane Virus replication by disrupting calcium-dependent steps of infection [142,143]. Notably, immunoglobulin-based interventions guided by HIE assays have successfully cleared chronic HuNoV infection in immunodeficient patients [144].

#### 3.2.2. Rotavirus

Rotavirus (RV) is a dsRNA virus belonging to the *Reoviridae* family and was historically the leading cause of severe diarrheal disease in children and immunocompromised individuals prior to widespread vaccine implementation. It remains a major global health burden, particularly in unvaccinated populations. RV primarily infects mature enterocytes of the small intestine [145,146,147,148,149]. Early organoid-based studies demonstrated productive RV infection, accompanied by cytopathic effects and induction of antiviral responses [150]. Mechanistic investigations using organoids in parallel with Caco-2 cells revealed that basal type I and type III interferon signaling suppresses RV replication, whereas inhibition of STAT1, STAT2, and IRF9 significantly increased viral replication. Exogenous treatment with IFN-α, IFN-β, or IFN-λ effectively reduced viral replication, supporting a central role for interferon-inducible interferon-stimulated genes (ISGs) in epithelial defense [151]. Consistent with this study, treatment of RV-infected HIOs with IL-22 enhanced epithelial proliferation and tissue repair, rather than directly inducing ISGs. This suggested a distinct yet complementary role of IFN-λ and IL-22 in conserving epithelial integrity during RV infection [152]. Further studies using HIOs infected with laboratory strains and primary RV isolates revealed a dependence on host metabolic pathways. Pharmacological inhibition of dihydroorotate dehydrogenase (DHODH) using brequinar or leflunomide, thereby blocking pyrimidine biosynthesis, significantly suppressed RV replication by limiting nucleotide availability [94]. Similarly, inhibition of guanosine synthesis with mycophenolic acid (MPA) resulted in approximately 99% reduction in viral RNA production. Additionally, the antidiabetic drug metformin hydrochloride suppressed RV replication in HIOs, Caco-2 cells, and murine models [153]. Transcriptomic analyses of HIEs infected with HuNoV, RV, and astrovirus identified a shared antiviral milieu characterized by type I interferon responses, ISG induction, and virus-specific signaling pathways [154]. It was also shown that lentiviral transduction of HIOs to express GCaMP6s enabled real-time visualization of calcium signaling dynamics during RV replication, further illustrating the versatility of organoid platforms for mechanistic virology studies [155].

#### 3.2.3. SARS-CoV-2

SARS-CoV-2 primarily infects the nasal epithelial lining and the respiratory tract, but viral tropism is also evident in the GI tract, as demonstrated by detection of viral RNA in feces and intestinal tissues [156,157]. The gut has been implicated as a key secondary site of infection, supported by reports of diarrhea and fecal shedding during COVID-19 [158,159]. Using ASC-derived HIEs and hPSC-derived intestinal organoids, it was demonstrated that enterocytes are susceptible to SARS-CoV-2, with these models exhibiting productive viral replication and cytopathic effects [160,161]. Organoid models derived from bat species and HIOs further provided insights into cross-species susceptibility and helped elucidate viral tropism and host-range barriers [162,163,164]. These organoid models have enabled investigation of multiple stages of the viral replication cycle, demonstrating the requirement of angiotensin-converting enzyme 2 (ACE2) and transmembrane serine protease 2 (TMPRSS2) for viral attachment and entry. They also revealed that infectivity and receptor usage are influenced by spike protein variation, with mutations such as R403/T403 modulating ACE2-dependent infection in lung and intestinal organoids. Additional studies showed that ACE2 expression is regulated by the farnesoid X receptor (FXR) and identified the lipolysis-stimulated lipoprotein receptor (LSR) as a critical entry mediator [165,166,167].

CRISPR-edited organoid models demonstrated that Omicron and other coronavirus variants continue to utilize TMPRSS2 for entry, providing mechanistic insight into altered transmissibility and tropism among VOCs [168,169]. Several studies using HIOs have extended beyond viral entry mechanisms. One investigation identified heat shock protein 90-beta (Hsp90β) as essential for stabilization of the coronavirus nucleoprotein; inhibition with 17-AAG suppressed replication of SARS-CoV, MERS-CoV, and SARS-CoV-2, highlighting Hsp90 as a potential antiviral target [170]. More recently, HIOs have been applied in translational contexts to explore prophylactic and therapeutic strategies. For example, an α-dystroglycan recombinant fragment inhibited SARS-CoV-2 replication, while MK-2206 (an Akt inhibitor) enhanced autophagy flux, limiting viral entry and preserving epithelial barrier integrity. Furthermore, intestinal organoids incorporating macrophages, termed macrophage-augmented organoids (MaugOs), were developed to study viral replication and evaluate immunomodulatory interventions against SARS-CoV-2 [171,172,173]. Finally, ESCs-derived HIOs were used to assess gut immune protection, demonstrating that serum from vaccinated individuals or those with prior exposure neutralized the WA01 strain, whereas reduced efficacy was observed against the Delta and Omicron variants [174]. The same organoid model was also employed to investigate TGEV-induced inflammation via the RIG-I/NF-κB/HIF-1α/glycolysis axis [175]. Moreover, HIF-1α was associated with enhanced viral replication and concomitant suppression of type I and type III interferon responses, suggesting that HIF-1α is a potential antiviral target [176].

#### 3.2.4. Enterovirus

Enterovirus A71 and other Picornaviruses, such as coxsackievirus B2 and poliovirus type 3, have been studied using HIOs. These organoid models demonstrated susceptibility to viral replication, while EV-D68 did not replicate productively [177]. Fetal human colon-derived organoids were also employed to study the replication kinetics of EV-A71, Coxsackievirus B3, and Echovirus 6, revealing approximately 10-fold higher viral replication than in 2D cultures. Further analyses identified goblet cells as the primary sites of viral replication [178]. Mechanistic studies using organoids showed that EV-A71 replication kinetics were strain dependent, with glutamine at position 145 of VP1 serving as a critical determinant of infectivity [179]. EV-A71 was released from enterocytes in a non-lytic manner following replication, involving the exosome pathway [180]. Differentiated HIOs were also used to investigate the role of the upstream open reading frame (uORF) protein in echovirus 7 and poliovirus 1. Knockout of the uORF protein resulted in attenuation during late stages of viral replication, indicating that this protein is essential for efficient viral shedding [181]. In another study, the Rock inhibitor GSK269962A suppressed EV-A71 replication, identifying Rock1 as a crucial and novel host factor for viral propagation [182].

Additional studies demonstrated that several vascular endothelial growth factor receptor (VEGFR) inhibitors exhibit potent antiviral activity, implicating VEGFR2 as another host factor required for EV-A71 replication, potentially mediated through the TSAd–Src–PI3K–Akt signaling pathway [183]. Leveraging HIOs, combination therapy with pleconaril, rupintrivir, and remdesivir was evaluated against multiple enteroviruses, including echovirus 1, echovirus 6, echovirus 11, coxsackievirus B5, and enterovirus A71, showing marked antiviral efficacy [184]. Beyond human organoids, mouse intestinal organoids demonstrated that EV-A71 replication induces store-operated Ca^2+^ entry (SOCE) via activation of the STIM/Orai1 complex, with viral replication occurring in an SOCE-dependent manner, thereby facilitating efficient viral propagation [185]. In a related investigation, “apical-out” porcine intestinal organoids were used to study senecavirus infection, revealing initial infection of enterocytes followed by sequential spread to other cell types, induction of stress granules, and activation of innate immune responses [186].

#### 3.2.5. Transmissible Gastroenteritis Virus (TGEV)

Transmissible gastroenteritis virus (TGEV), a positive-sense single-stranded RNA (+ssRNA) virus of the *Coronaviridae* family, played a pivotal role in the development and use of PIOs [187]. Apical-out PIO models were employed to assess viral infectivity and host immune responses, demonstrating robust TGEV replication accompanied by upregulation of IFN-α, IFN-λ1, and inflammatory mediators, including TNF-α and IL-6 [188]. In one study, PIOs were used to investigate epithelial regeneration during TGEV infection, revealing activation of the Wnt/β-catenin pathway and increased expression of intestinal stem cell (ISC) self-renewal markers following viral challenge [189]. By optimizing HIO growth conditions, a long-term PIO culture system was established, enabling sustained experimental modeling and continuous release of TGEV progeny [190]. To examine virus-mucus-epithelium interactions, a mucus layer was generated in PIOs using an air-liquid interface (ALI) configuration. Subsequent TGEV infection demonstrated reduced viral infectivity, with mucin 2 (MUC2) exhibiting antiviral activity and sialic acid contributing to viral inhibition [191]. Thapsigargin (TG), an ER stress inducer and an oral antiviral drug candidate, was reported to inhibit TGEV replication in a study using PIOs infected with TGEV [192]. Furthermore, PIOs were generated from Wuzhishan miniature pigs (WZS) exhibited susceptibility to TGEV, and transcriptomic analyses revealed induction of antiviral and inflammatory responses [193].

#### 3.2.6. Porcine Epidemic Diarrhea Virus (PEDV)

Porcine epidemic diarrhea virus (PEDV) is another member of the family *Coronaviridae* [194]. Organoid-based studies on PEDV have primarily focused on host factors and therapeutic interventions. PIOs infected with PEDV supported sustained viral replication and progeny release. Additionally, RNA-seq analysis revealed activation of antiviral signaling pathways and immune responses [190]. Differentiated porcine enteroid monolayer cultures (PEMCs) in Matrigel were used to assess differences in PEDV susceptibility across intestinal segments. The results showed that all segments were susceptible to PEDV, with jejunum-derived PEMCs exhibiting significantly higher viral replication [195]. Furthermore, PIOs derived from the small intestine were used as a platform to validate genome-wide CRISPR/Cas9 screening to identify key host factors involved in PEDV infection, leading to the identification of IFITM1 as a host factor associated with enhanced PEDV entry [196].

In addition, intestinal barrier integrity during PEDV infection was investigated using intestinal organoids, highlighting the role of long noncoding RNAs (lncRNAs). Knockdown of lncRNA446 increased PEDV replication, and disruption of tight junctions was observed, as lncRNA446 regulates tight junction integrity by preventing Alix ubiquitination [197]. Milk small extracellular vesicles (sEVs) were found to suppress PEDV infection in PIOs; further investigation revealed that cargo miRNAs, miR-let-7e and miR-let-27b, exert antiviral functions [198]. The coexistence of *Trichinella spiralis* and PEDV was modeled in PIOs, demonstrating that *T. spiralis* excretory/secretory antigens (TsES) enhanced PEDV-induced inflammation and damaged the mucosal barrier in organoids, despite TsES contributing to PEDV replication [199].

#### 3.2.7. Middle East Respiratory Syndrome Coronavirus (MERS-CoV)

In 2017, the human gastrointestinal tract was hypothesized to serve as an alternative route of MERS-CoV infection, which was subsequently investigated using HIOs. These organoids were found to be susceptible to MERS-CoV, as evidenced by robust viral replication [200]. In addition, intestinal organoids derived from *Rousettus aegyptiacus* were established to mechanistically explore bat adaptability to zoonotic viruses. This model was challenged with MERS-CoV and Marburg virus, revealing heightened expression of immune effectors, including IFN-ε, interferon-stimulated genes (ISGs), and type III interferon IFN-λ. Notably, this response exhibited self-amplification, conferring a strong antiviral state in a virus-independent manner [201].

#### 3.2.8. Human Astrovirus (HAstV)

Human astrovirus (HAstV) is a positive-sense single-stranded RNA (+ssRNA) virus belonging to the family *Astroviridae* [202]. To validate findings from genome-wide CRISPR–Cas9 screening, intestinal organoids (IOs) were used to identify the cellular receptor for HAstV. Deletion of the Fc gamma receptor and transporter (FCGRT) and β2-microglobulin (B2M), which together encode the neonatal Fc receptor (FcRn), rendered IOs resistant to HAstV infection, thereby establishing FcRn as the functional receptor for HAstV [203]. Furthermore, scRNA-seq analysis of HAstV-infected HIOs was performed to unravel immune dynamics across individual cell populations. The results demonstrated that HAstV can infect all major intestinal cell types and induces specialized antiviral transcriptional reprogramming, with distinct basal gene expression patterns observed across different cell populations [204].

#### 3.2.9. Mammalian Reovirus (MRV)

Mini-gut organoids were used to delineate the roles of Type I and Type III Interferons (IFNs) in protecting the human gut against mammalian reovirus (MRV) infection. Upon viral challenge, human intestinal epithelial cells (IECs) secreted only type III IFN into the supernatant, and the antiviral activity mediated by type III IFNs was strongly dependent on the mitogen-activated protein kinase (MAPK) signaling pathway. These findings suggest that Type III IFN constitutes a spatially restricted frontline antiviral response in the human gut [205]. Separately, porcine intestinal organoids (PIOs) were established and infected with mammalian orthoreovirus Type 3 (MRV3). Infected PIOs exhibited delayed proliferation, structural disruption, and altered gene expression associated with intestinal epithelial function and antiviral responses [206].

#### 3.2.10. Avian Influenza Virus (AIV)

Mouse intestinal organoids containing crypts and villi were generated from ISCs and used to investigate interactions with H9N2 avian influenza virus. The study demonstrated that H9N2 viral genes were detectable, with peak levels observed at 48 h post-infection (hpi). Infection caused significant intestinal damage, including impaired intestinal stem cell proliferation and differentiation, as well as loss of Paneth cells [207]. In parallel, chicken intestinal organoid (CIO) systems were employed to examine replication dynamics and innate immune responses to low-pathogenic avian influenza viruses (LPAIVs), including the endemic H6N1 strain and the Eurasian H9N2 strain. The results showed that apical-out organoids, mimicking natural exposure, elicited robust interferon-stimulated gene (ISG) responses that effectively controlled H6N1 replication, whereas “basal-out” organoids exhibited weaker upstream interferon responses during H9N2 infection [208].

#### 3.2.11. Other Enteric Viruses

For other enteric viruses, several studies have employed organoid models to define host range and pathogenicity. PIOs were used to investigate the cross-species transmission potential of Human coronavirus OC43 (HCoV-OC43), where viral inoculation demonstrated high susceptibility, infectious virus production, and widespread cellular death [209]. Bovine enteroids established from bovine ileum served as an *in vitro* replication system for Bovine Coronavirus (BCoV) and were found to be permissive to infection, with BCoV inducing downregulation of immune-related genes, including CXCL-3, MMP13, and TNF-α [210]. Chicken intestinal organoid monolayers were used to study the attenuated response to porcine deltacoronavirus (PDCoV) infection in chickens, where abortive PDCoV infection activated the Wnt/β-catenin pathway, enhancing intestinal stem cell (ISC) self-renewal and accelerating epithelial regeneration, thereby contributing to resistance against PDCoV infection [211]. Rabbit intestinal organoids (RIOs) were also propagated and challenged with Rabbit calicivirus Australia-1; however, despite testing multiple inoculation conditions, no viral replication was observed [212].

Comparative analysis across enteric viruses using intestinal organoids demonstrates both shared requirements, such as mature enterocyte infection and interferon-mediated restriction, and virus-specific dependencies on host genetics, bile acids, metabolic pathways, and immune modulation. These findings illustrate how epithelial context shapes divergent replication and pathogenesis strategies. The introduction of intestinal organoids has fundamentally transformed experimental access to enteric virus biology, most notably for human norovirus. In contrast to immortalized cell lines, which lack key epithelial differentiation states, stem cell-derived enteroids recapitulate host genetic determinants, including histo-blood group antigens and FUT2 expression, that govern viral susceptibility. This physiological fidelity enabled sustained HuNoV replication *in vitro* for the first time, overcoming a long-standing barrier in the field and directly linking patient-derived observations with mechanistic investigation. Key findings from enteric virus studies using organoid models are summarized in Table 2.

### 3.3. Liver Organoids for Hepatotropic Viruses

Often referred to as mini-livers, liver organoids (LOs) are 3D structures that recapitulate the cytoarchitecture and key functional properties of the human liver. Like other organoid systems, they are generated using two main approaches: expansion of adult stem cells or differentiation of pluripotent stem cells. These organoids exhibit hepatocyte-like functions and are permissive to infection by multiple hepatotropic viruses, enabling productive viral replication and providing physiologically relevant platforms for dissecting viral life cycles and host responses.

#### 3.3.1. Hepatitis B Virus (HBV) and Hepatitis D Virus (HDV)

Liver organoids have emerged as physiologically relevant platforms for modeling chronic hepatitis virus infections, particularly HBV and its satellite virus HDV, which depends on HBV surface antigens for entry and propagation. hIPSC-derived LOs comprising endodermal, mesenchymal, and endothelial lineages have been successfully used to establish HBV infection in 3D microwell systems, where viral challenge induced hepatic dysfunction and dysregulation of liver-specific gene expression programs [213]. Complementary studies using patient-derived LOs further enabled investigation of HBV-driven hepatocarcinogenesis, providing *ex vivo* platforms for personalized therapeutic assessment [214,215,216]. In one model, LOs generated from healthy donors were infected with recombinant HBV or patient-derived serum, leading to the formation of covalently closed circular DNA (cccDNA) and the production of infectious virions. Transcriptomic profiling of infected organoids revealed early cancer-associated gene signatures that clustered with hepatocellular carcinoma cohorts, linking HBV replication directly to oncogenic transcriptional reprogramming [217]. These platforms have also facilitated antiviral evaluation, including orally administered agents targeting cellular inhibitors of apoptosis, which selectively eliminated infected hepatocytes and promoted clearance of episomal HBV genome [218]. Subsequent methodological refinements further optimized recombinant HBV infection efficiency in human LOs, improving experimental reproducibility and scalability [219,220].

Beyond hepatocyte-derived organoids, intrahepatic cholangiocyte organoids (ICOs) have been explored as personalized infection models using HepAD38-derived and plasma-derived HBV. ICOs supported productive HBV replication with pronounced donor-dependent variation in viral dynamics and host transcriptional responses. Notably, HBV infection failed to induce ISGs, despite preserved responsiveness to exogenous interferon, highlighting intrinsic viral immune evasion mechanisms [221]. Additional studies demonstrated that HBV transcription disrupts hepatocyte developmental programs by activating DNA repair pathways and enhancing glycolytic metabolism, molecular features closely associated with hepatocellular carcinoma progression [222]. Within this HBV-dependent framework, HDV pathogenesis has also been interrogated using organoid-based approaches. Ubiquitinated small hepatitis D antigen (Ub-S-HDAg) was shown to promote dendritic cell maturation and CD8^+^ T cell activation via JAK/STAT signaling, correlating with enhanced antiviral immunity and reduced viral load. These findings highlight how HDV modulates host immune pathways within HBV-infected hepatic environments, underscoring the utility of liver organoids for dissecting complex viral co-infections and immune interactions [223].

#### 3.3.2. Hepatitis C Virus (HCV)

Liver organoids have also been used to investigate polarity-dependent mechanisms of viral entry and host immune responses to hepatitis C virus (HCV). Studies examining interactions between host cell receptors and HCV-like particles (HCV-VLPs) in polarized LOs versus conventional 2D cultures demonstrated that productive receptor engagement occurred exclusively in polarized organoids, underscoring the importance of epithelial architecture for HCV entry [224,225]. Complementary single-particle imaging in 3D polarized hepatoma models further revealed that HCV entry is a multistep process: the virus initially engages scavenger receptor class B type 1 (SR-B1) and CD81 at the basolateral membrane via actin-driven movement, followed by accumulation at tight junctions and subsequent internalization through clathrin-mediated endocytosis [226]. Multicellular LOs incorporating Kupffer-like cells have also been used to model HCV-associated liver disease progression, in which infection was linked to lipid accumulation and enhanced lipogenic gene signatures in host cells, recapitulating key metabolic features of HCV pathogenesis [227]. Beyond innate responses, ASC-derived Los, when integrated with microfluidic platforms, enabled co-culture with HLA-matched CD8^+^ T cells. Upon stimulation with HCV NS3 peptides and patient-derived CD8^+^ cells, this system facilitated targeted immune-mediated injury of infected LOs, providing a physiologically relevant platform for studying adaptive immune responses during HCV infection [228].

#### 3.3.3. Hepatitis E Virus (HEV)

Fetal and adult LOs containing hepatocyte and cholangiocyte populations have been shown to support the complete HEV replication cycle and viral shedding when cultured as polarized monolayers. Using these systems, drug screening approaches identified brequinar and homoharringtonine as effective inhibitors of HEV replication [229]. Early innate immune responses were also observed, with HEV RNA triggering IRF3- and IRF7-dependent interferon signaling in primary LOs generated from hepatocyte-like cells cultured on inverted colloidal crystal scaffolds.

Beyond mechanistic investigations, liver organoids have emerged as robust platforms for antiviral discovery against HEV. Drug repurposing and targeted screening strategies using human liver organoids identified multiple host-directed inhibitors, including niclosamide, brequinar, homoharringtonine, vidofludimus calcium, and pyrazofurin, which suppress HEV replication by modulating NF-κB signaling and pyrimidine biosynthesis pathways. Notably, these studies demonstrated antiviral efficacy against HEV variants associated with ribavirin treatment failure, underscoring the translational relevance of organoid-based platforms for identifying therapeutic options for chronic and drug-resistant HEV infections. Collectively, these findings establish liver organoids as a powerful system for modeling HEV replication dynamics and innate antiviral responses in a human-relevant hepatic context.

#### 3.3.4. Dengue Virus (DENV)

hPSC-derived LOs have been employed to investigate dengue virus infection and associated hepatic pathology. These models were susceptible to DENV-2 infection, exhibiting extensive cell death and structural disruption that resembles severe dengue pathology. Single-cell RNA sequencing revealed that proliferating hepatocyte-like cells constituted the predominant infected population and demonstrated pronounced mitochondrial injury accompanied by alterations in cellular composition. Subsequent drug screening studies identified oxyresveratrol (ORES) and omaveloxolone (RTA403) as antiviral compounds in this system. Both agents activated the NRF2 pathway, reduced oxidative stress, and preserved mitochondrial function [188].

#### 3.3.5. Liver Organoids to Study Oncolytic Viruses

LOs are increasingly being utilized to investigate oncolytic viruses (OVs) and their therapeutic potential in HCC. However, oncolytic adenoviruses (OAs) face significant challenges, including rapid hepatic clearance and low liver tropism of commonly used vectors such as Ad5 [230,231]. In contrast, third-generation oncolytic HSV-1 (T-01) has demonstrated enhanced T cell-mediated antitumor efficacy against HCC in organoid-based models [232]. Similarly, coxsackievirus A21 (V937), in combination with IFN-γ and PD-1 blockade, promoted immune activation and oncolysis in HCC organoids co-cultured with PBMC [233]. Beyond human tumor models, lagoviruses, including RHDV1, RHDV2, and RHDVa-K5, have been evaluated in hepatobiliary organoids derived from European rabbits and brown hares. These studies demonstrated that inhibition of interferon signaling increased viral susceptibility, further underscoring the utility of liver organoids for dissecting host antiviral responses and oncolytic virus biology [234,235].

#### 3.3.6. Non-Canonical Hepatotropic Viruses and Liver Involvement

Beyond classical hepatitis viruses, liver organoids have enabled investigation of additional liver-tropic and systemically disseminating viruses that contribute to hepatic pathology. Human pluripotent stem cell-derived liver organoids were shown to be highly permissive to SARS-CoV-2 infection, exhibiting productive viral replication and robust induction of inflammatory and chemokine signaling pathways, consistent with liver injury observed in severe COVID-19 patients [236]. Single-cell transcriptomic profiling of infected liver organoids further revealed extensive cytokine signaling and bystander inflammatory responses, implicating IL-6-mediated pathways in virus-associated hepatic immune activation [237]. Complementary studies using liver organoid-derived intrahepatic bile duct cells demonstrated efficient SARS-CoV-2 replication in cholangiocytes, supporting direct cytopathic effects as a contributing mechanism underlying COVID-19-associated liver dysfunction [238].

In addition to coronaviruses, liver organoids have been applied to dissect host dependency factors for acute hepatotropic viruses. A genome-wide CRISPR-Cas9 screen validated in human liver organoids identified UFMylation machinery and TRAMP-like complexes as essential host factors required for hepatitis A virus translation, establishing liver organoids as powerful platforms for uncovering host-directed antiviral targets [239]. Furthermore, drug repurposing screens conducted in liver organoid models identified host-targeting inhibitors that suppress viral replication by blocking pyrimidine biosynthesis, including compounds effective against treatment-refractory hepatitis virus strains, reinforcing the translational relevance of organoid-based antiviral discovery pipelines [240,241]. Collectively, these studies highlight the versatility of liver organoids in modeling diverse hepatotropic and liver-tropic viral infections, enabling mechanistic dissection of viral replication, host signaling pathways, and antiviral intervention strategies within a physiologically relevant human hepatic context [242].

Across hepatotropic and oncolytic viruses, liver organoid models reveal recurring patterns of polarity-dependent epithelial entry into hepatocytes and cholangiocytes, accompanied by innate immune activation, while also enabling direct comparison of strain-dependent differences in replication efficiency, tissue damage, and antiviral sensitivity under physiologically relevant exposure conditions. Liver organoids provide an experimental context that more closely reflects hepatic physiology than hepatoma cell lines, particularly by preserving epithelial polarity and metabolic activity that shape viral infection outcomes. This is especially evident in hepatitis C Virus studies, where receptor engagement at polarized membranes, such as interactions involving SR-B1 and CD81, can be resolved with clarity not achievable in non-polarized 2D cultures. In parallel, the capacity of liver organoids to sustain covalently closed circular DNA during hepatitis B virus infection enables direct interrogation of viral persistence and chronicity-associated molecular signatures that are typically lost in conventional *in vitro* models. Table 3 summarizes the application of liver organoids in studying hepatotropic and oncolytic viruses.

### 3.4. Lung Organoids for the Study of Respiratory Viruses

Lung organoids, also known as airway organoids (AOs) or alveolar organoids (ALOs), are 3D self-organizing structures that recapitulate the cellular composition and cytoarchitecture of the human respiratory tract [244]. A key strength of lung organoid models is their ability to support viral infection via the apical epithelial surface, thereby closely mimicking natural respiratory exposure. This enables physiologically relevant assessment of viral tropism, cytopathic effects, disruption of epithelial barrier, and innate immune responses [245,246,247].

#### 3.4.1. SARS-CoV-2

Lung organoids have been instrumental in elucidating SARS-CoV-2 tropism, pathogenesis, and therapeutic responses. hESC-derived airway and alveolar organoids supported productive viral replication, with infection predominantly targeting ciliated cells, club cells, and alveolar type 2 (AT2) cells along the proximal-distal lung axis. RNA-seq analysis further revealed robust immune activation accompanied by suppression of metabolic and lipid pathways [248]. Similarly, ASC-derived lung organoid comprising both proximal and distal epithelial populations recapitulated transcriptomic signatures observed in COVID-19 patients. In these models, proximal airway cells sustained viral proliferation, while hyperinflammatory responses were associated with AT2 differentiation [249].

Airway organoids have also been used for antiviral screening, identifying several candidate therapeutics. Remdesivir significantly reduced viral load [248], polyclonal antibodies prevented tissue injury and viral progression, whereas atorvastatin inhibited viral attachment and entry [250]. In addition, viral replication was suppressed in a dose-dependent manner following treatment with immunosuppressive agents, including mycophenolic acid, 6-thioguanine, tofacitinib, and filgotinib [251]. Nasal organoids cultured at an air-liquid interface further captured hallmark pathological features of infection, including ciliary loss and mucus overproduction [252].

#### 3.4.2. Influenza Virus

Human airway organoids derived from lung stem cells have provided physiologically relevant platforms for investigating influenza A virus (IAV) tropism and replication. These systems recapitulate key features of *ex vivo* bronchus cultures, enabling assessment of viral replication dynamics and cell-type specificity for pandemic H1N1pdm and avian influenza strains, including H7N9, H5N1, and H5N6. H1N1pdm and H7N9 exhibited significantly higher replication efficiency compared with H5N1 and preferentially infected ciliated and goblet cells [253]. Evaluation of emerging strains using differentiated airway organoids in 3D and air-liquid interface (ALI) cultures further demonstrated that human-infective H7N9/Ah replicated more efficiently than the human-adapted H7N2 strain [21]. Prolonged H5N1 infection induced fibrotic remodeling in human airway organoids, characterized by increased expression of α-SMA, collagen, and fibronectin [254]. Swine IAV virulence has also been assessed using epithelial cultures derived from porcine airway organoids under ALI conditions. The H3N2 strain caused greater epithelial barrier disruption and tight junction damage compared with H1N2 or H1N1, illustrating the utility of organoid models for comparative virulence screening among influenza strains [255].

Comparative analyses across multiple studies demonstrated that viral replication kinetics, cell-type tropism, and cytokine induction profiles observed in airway organoids closely mirror those seen in *ex vivo* human bronchial explants, validating organoids as physiologically relevant alternatives for studying both seasonal and zoonotic influenza viruses. While H1N1pdm and H7N9 replicate efficiently in ciliated and mucus-secreting epithelial cells, highly pathogenic H5N1 induces disproportionately strong inflammatory responses despite lower replication efficiency, highlighting strain-specific differences linked to pathogenic potential [253]. Differentiated airway organoids have also proven reliable for predicting human infectivity of emerging influenza viruses, with human-adapted strains replicating more robustly than poorly human-infective avian or swine isolates [21].

Beyond viral replication, airway organoids have enabled identification of host regulatory factors, such as airway serine proteases and their endogenous inhibitors, which govern hemagglutinin activation and viral spread, processes that are difficult to resolve in conventional monolayer cultures [256]. Together, these findings underscore the capacity of airway organoids to capture polarity-dependent infection, interferon compartmentalization, and strain-specific virulence within a single human-relevant experimental platform.

#### 3.4.3. Human Respiratory Syncytial Virus (HRSV)

Human Respiratory Syncytial Virus (HRSV) or simply Respiratory Syncytial Virus (RSV) is a negative-sense single-stranded RNA (−ssRNA) virus belonging to the family *Pneumoviridae* and is a leading cause of pediatric respiratory infection [257]. Lung organoids modeling first-trimester fetal lung development were injected with recombinant RSV-A2, resulting in dose- and time-dependent viral propagation, increased CC10 expression in a dose-dependent manner, and the disruption of the F-actin cytoskeleton [258]. Maturity-dependent susceptibility was demonstrated by comparing immature fetal lung organoids with mature induced airway organoids, revealing that mature airway epithelial cells restricted RSV replication more effectively by mounting stronger innate immune responses than their immature counterparts [259]. Mechanistic studies using differentiated human airway organoids identified insulin-like growth factor 1 receptor (IGF1R) as a key mediator of RSV entry, whereby IGF1R activation triggered signaling cascades that recruited the co-receptor nucleolin to the cell surface via protein kinase C zeta (PKCζ) [260]. An apical-out airway organoid model was further developed to expose CX3CR1^+^ ciliated cells, enabling direct viral access to physiologically relevant target populations. This configuration proved effective for high-throughput neutralization assays, accurately capturing the activity of both F- and G-specific antibodies, and provides a robust platform for investigating protective immune responses to RSV [246].

#### 3.4.4. Parainfluenza Virus (PIV) and Seasonal Coronaviruses

Human airway organoids (hAOs) have been effectively used to model infection dynamics and drug responses for seasonal coronaviruses, including 229E, OC43, and NL63. These viruses exhibited more than 10-fold higher replication at 33 °C than at 37 °C, consistent with their preference for the cooler upper airway environment [261]. Treatment with molnupiravir and remdesivir significantly reduced viral replication in a dose-dependent manner. For human parainfluenza viruses (HPIVs), organoid-derived bronchial and tracheal air-liquid interface cultures proved critical for maintaining the genetic integrity and phenotypic characteristics of all four HPIV types, preventing the adaptive mutations commonly observed in conventional cell lines [262].

#### 3.4.5. Adenovirus

hESC-derived airway and alveolar organoids have been used to compare HAdV-3 with the more virulent HAdV-55, demonstrating that HAdV-55 replicates more efficiently and exhibits broader tropism toward alveolar stem cells and airway epithelial populations [263]. These models have also been used for antiviral evaluation, in which cidofovir demonstrated efficacy against HAdV infection [263].

Across respiratory viruses, lung organoid models reveal recurring patterns of apical epithelial entry, tropism toward ciliated and secretory cells, and innate immune activation, while enabling direct comparison of strain-dependent differences in replication efficiency, tissue damage, and antiviral sensitivity under physiologically relevant exposure conditions. Lung organoid systems provide a level of epithelial complexity that is difficult to maintain in conventional airway monolayers, preserving ciliated, secretory, and alveolar populations over extended culture periods. This has allowed respiratory viruses such as SARS-CoV-2 and influenza to be examined within a spatial framework that reflect the proximal-distal organization of the human airway. Moreover, apical-out configurations and air-liquid interface cultures reproduce physiological exposure conditions, enabling interrogation of mucociliary clearance, epithelial barrier disruption, and localized immune responses that are poorly modeled under submerged 2D systems. A summary of respiratory viruses investigated using lung organoid models is presented in Table 4.

### 3.5. Kidney Organoids

Kidney Organoids are self-assembled 3D structures that mimic the complex architecture and cellular diversity of the native human kidney. These models generate multiple nephron segments along with endothelial and stromal components, providing physiologically relevant platforms for renal biology and disease modeling [264,265]. Similarly to other organoid systems, kidney organoids bridge critical gaps between animal models and conventional 2D cultures by enabling direct investigation of human-specific host–pathogen interactions in infectious disease contexts [266,267]. Recent studies have demonstrated that kidney organoids support productive viral infection and exhibit robust virus-induced renal pathology [268]. Consequently, kidney organoids serve as complementary and translationally relevant models for investigating viral kidney involvement and evaluating antiviral strategies in human-specific settings [269,270].

#### 3.5.1. SARS-CoV-2

In addition to the lungs, brain, and gastrointestinal tract, SARS-CoV-2 also infects renal tissue, facilitated by expression of ACE2 and TMPRSS2 in proximal tubular epithelial cells. Kidney organoids (KOs) recapitulate this susceptibility, supporting productive viral replication, apoptosis, and morphological alterations. Transcriptomic analyses of SARS-CoV-2-infected KOs revealed upregulation of interferon signaling pathways that mirrored urinary proteomic signatures observed in critically ill COVID-19 patients. Preclinical therapeutic studies using KOs demonstrated that viral infection could be blocked by novel spike-binding peptides and neutralized by a long-acting soluble ACE2 variant (ACE2 1-618-ABD) [271,272]. Further investigations showed that combinatorial treatment with remdesivir and soluble ACE2 significantly enhanced the therapeutic window against SARS-CoV-2 infection [273].

#### 3.5.2. Mpox Virus (MPXV)

Mpox Virus is a dsDNA virus belonging to the family *Poxviridae* [274,275]. Kidney organoid studies demonstrated susceptibility to MPXV infection, with organoids responding favorably to antiviral treatment, further highlighting their utility for modeling viral renal involvement and therapeutic evaluation [276].

Although still limited in number, kidney organoid studies across diverse viral families demonstrate a shared capacity to model proximal tubule tropism, interferon-driven responses, and virus-induced renal injury, supporting their emerging role in elucidating mechanisms of viral nephropathology and therapeutic intervention. Kidney organoid platforms capture nephron-level cellular diversity largely absent from conventional renal cell lines, enabling the resolution of virus–host interactions at the segment-specific level. Using these systems, SARS-CoV-2 infection has been localized predominantly to ACE2- and TMPRSS2-expressing proximal tubular cells, producing injury patterns that parallel urinary proteomic signatures reported in critically ill COVID-19 patients. The ability of kidney organoids to reproduce clinically relevant features of viral nephropathy in an *ex vivo* setting underscores their added value over traditional *in vitro* approaches. Table 5 summarizes the viral infection studies conducted using kidney organoid models.

## 4. Assembloids: Multicellular and Multi-Organoid Platforms for Studying Viral Pathogenesis

Assembloids represent an advanced evolution of organoid technology, generated through the controlled integration of multiple region-specific organoids or distinct cellular compartments to model inter-tissue communication, circuit formation, and complex host–pathogen interactions. Unlike single-organoid systems, assembloids enable investigation of viral pathogenesis within a spatially and functionally interconnected human tissue context, allowing assessment of viral spread, cell-to-cell transmission, bystander effects, and circuit-level dysfunction that cannot be resolved in isolated cultures.

Recent virology-focused studies have demonstrated the unique utility of assembloids in modeling human-restricted neurotropic viral infections. Human spinal cord-muscle and brain-peripheral tissue assembloids have been used to model poliovirus, EV-D68, and EV-A71 infection, revealing virus-specific differences in cellular tropism, kinetics of neuronal injury, and mechanisms leading to functional paralysis despite shared clinical outcomes. These systems captured convergent loss of neuromuscular activity driven by divergent viral strategies, an insight not achievable in monolayer cultures or single neural organoids [277]. Assembloids have further enabled circuit-level interrogation of viral neuropathogenesis, demonstrating that viral infection can disrupt neuronal connectivity and network synchrony independent of overt cytolysis. By preserving functional synaptic integration across regions, assembloids permit direct assessment of how viruses perturb information flow, neurotransmission, and emergent network behavior, critical dimensions of neurovirology that are poorly modeled in conventional systems [278,279]. Beyond neurotropic viruses, assembloid platforms incorporating immune-epithelial interfaces have been applied to study infection-induced inflammatory responses, including macrophage-epithelium interactions and paracrine signaling cascades that amplify tissue damage. These models reveal how immune components modulate viral permissiveness, persistence, and host injury, phenomena obscured in immune-deficient organoid systems [280].

From a technological perspective, advances in regionally patterned and inter-regional assembloids have improved reproducibility, cellular maturation, and functional complexity, enabling long-range projections, polarized signaling, and directional migration of infected cells. Such features are particularly relevant for studying viral dissemination across tissue boundaries, including CNS entry routes, axonal transport, and secondary organ involvement [281]. Importantly, assembloids are increasingly integrated with microfluidic platforms, vascularized components, and organ-on-chip technologies, further enhancing their relevance for modeling viral transmission dynamics, endothelial infection, and barrier-crossing events. These hybrid systems offer precise control over nutrient gradients, oxygenation, and flow, facilitating studies of viral access routes and systemic spread under near-physiological conditions [282,283].

Looking forward, assembloid-based virology holds substantial promise for addressing unresolved questions in viral pathogenesis, including multi-organ crosstalk, immune-mediated bystander injury, persistent infection, and post-viral sequelae. The incorporation of patient-derived cells, immune components, and vascular networks positions assembloids as powerful platforms for translational antiviral discovery and precision virology, complementing and, in some contexts, surpassing traditional organoid, air-liquid interface, and animal models [284,285]. Furthermore, continued integration of assembloids with advanced microfluidic and organ-on-chip systems is expected to expand experimental capabilities by enabling controlled interrogation of viral dissemination and barrier traversal under physiologically relevant flow conditions, thereby broadening the scope of human-relevant virology models [286].

## 5. Organ-on-Chip Applications in Virology

Organ-on-Chip (OoC) is emerging as an advanced, human-relevant alternative platform that narrows the gap between 2D culture and animal models to study viral infections and host responses [27,287,288,289]. Due to the incorporation of dynamic fluid flow, physiological mechanical cues, and tissue architecture, OoC models enable detailed investigation of viral replication stages, viral tropism, drug evaluation, and immune signaling [290,291,292,293]. For the study of respiratory viruses such as SARS-CoV-2 and influenza, respiratory OoC systems platforms were widely used. These studies revealed compartment-specific antiviral responses and differential interferon and chemokine signaling across airway and alveolar regions [291,293,294]

Integration of multiple tissue compartments in the OoC systems has further enhanced the modeling of systemic and indirect antiviral effects, as evidenced by lung-brain and multi-organ microphysiological platforms that captured inflammation-driven endothelial injury and extrapulmonary pathology following respiratory infection [291,292]. Placental OoC models have also been used to study enteric viruses and organ-specific viral infections [295]. Collectively, these studies lay the foundation for establishing organ-on-chip technologies as a next-generation, adaptable tool for understanding viral pathogenesis, evaluating antivirals, and advancing translational virology.

## 6. What Organoids Have Taught Us About Viruses: Conceptual Insights from Organoid-Based Virology

Although organoid-based virology studies are often organized by organ system or virus family, synthesis across these models reveals several unifying biological principles that transcend individual pathogens. Rather than serving merely as permissive infection systems, organoids function as hypothesis-generating platforms that uncover how tissue architecture, cellular differentiation, and local immune programs converge to shape viral pathogenesis. One of the most consistent insights emerging from organoid studies is the central role of epithelial polarity in governing viral entry, replication, and egress. In intestinal, airway, and hepatic organoids, polarized tissue organization dictates receptor accessibility and the spatial directionality of infection. This has been exemplified by norovirus dependence on apical histo-blood group antigens, apical entry of SARS-CoV-2 via ACE2, and basolateral interactions of hepatotropic viruses. Such polarity-dependent processes are poorly captured in conventional 2D cultures, underscoring a fundamental advantage of 3D organoid systems in resolving physiologically relevant infection routes.

A second unifying theme is the strong influence of cellular differentiation state on viral permissiveness and disease outcome. Across brain, intestinal, lung, and liver organoids, progenitor or less differentiated cell populations frequently exhibit heightened susceptibility to infection, whereas mature cells tend to mount more effective intrinsic antiviral responses. This principle is evident in the targeting of neural progenitor cells by the Zika virus, the preferential infection of differentiated enterocytes by rotavirus and norovirus, and the SARS-CoV-2 tropism for differentiated airway and alveolar epithelial cells. Organoids uniquely enable the coexistence of multiple developmental states within a single system, allowing differentiation-dependent viral dynamics to be dissected in ways not feasible in traditional models. Comparative analyses further clarify the contexts in which organoids offer distinct advantages over alternative platforms such as air-liquid interface cultures or short-term *ex vivo* tissues. While ALI systems remain well-suited for studying acute respiratory infection and mucociliary function, organoids are particularly powerful when experimental questions involve 3D architecture, multicellular interactions, viral latency, chronic infection, or long-term tissue remodeling. Importantly, organoid models have also revealed that innate immune responses are highly tissue- and cell-type specific rather than uniformly antiviral. Dominance of type III interferon responses in intestinal epithelia, attenuated interferon signaling during hepatotropic virus infection, and context-dependent inflammatory responses in neural organoids challenge immune paradigms derived from immortalized cell lines. These findings emphasize the necessity of studying antiviral immunity within appropriate tissue environments and caution against extrapolating universal immune mechanisms across organs.

Collectively, these conceptual insights position organoid-based virology as a critical bridge between reductionist cell culture systems and in vivo studies. By capturing physiologically relevant architecture, differentiation, and immune context, organoids advance the field beyond descriptive infection models toward mechanistic frameworks with direct implications for antiviral discovery, vaccine design, and prediction of tissue-specific disease outcomes.

## 7. Future Perspectives of Organoids in Virology

Organoid systems are increasingly evolving from purely descriptive infection models into platforms that allow more integrative analysis of virus–host interactions. An important future direction is to enhance system-level complexity by combining organoids with microfluidic and organ-on-chip approaches. Such integration enables dynamic perfusion, controlled mechanical stimulation, and limited inter-organ communication, which together improve tissue maturation and permit more physiologically relevant modeling of viral dissemination, systemic infection, and organ–organ crosstalk. The incorporation of immune components into organoid systems, including co-culture with innate and adaptive immune cells, autologous immune reconstruction, or the development of engineered immune niches, is expected to improve the capacity of these models to interrogate antiviral immunity, immune evasion strategies, and virus-induced immunopathology. Such immune-augmented organoids may be particularly informative for examining vaccine responses, post-infectious inflammatory processes, and immune-mediated tissue damage.

Future applications of organoid platforms are also likely to extend toward personalized and longitudinal infection modeling. Patient-derived organoids offer an opportunity to investigate how host-specific factors, such as genetic background, age, comorbidities, and prior immune exposure, influence viral susceptibility, disease progression, and therapeutic response over time. In parallel, incorporation of spatial organization, biomechanical cues, and extracellular matrix dynamics may further refine how viral entry, replication, and host signaling pathways are represented *in vitro*. Finally, the continued integration of organoid systems with single-cell multi-omic approaches, CRISPR-based genetic perturbation strategies, and scalable high-throughput screening platforms is expected to accelerate the identification of host factors and therapeutic targets. Collectively, these advances support the use of organoids as increasingly predictive preclinical models that connect mechanistic insights in virology with antiviral development, vaccine evaluation, and preparedness for future emerging and re-emerging viral threats.

## 8. Limitations and Challenges of Organoid Systems in Virology

Even though organoid technology has revolutionized virology by enabling detailed analysis of host–virus interactions, host factor modulation, and immune responses, several limitations continue to constrain full translational implementation.

A major challenge across organoid platforms is limited structural maturity and incomplete cellular diversity, restricting faithful recapitulation of in vivo organ function. Numerous PSC-derived organoids exhibit immature phenotypes, compromising architectural fidelity and physiological performance [296,297]. Furthermore, most organoids lack vascular perfusion and native stromal microenvironments, resulting in suboptimal oxygen and nutrient delivery and limiting long-term maintenance [298,299]. Recent methodological advances are beginning to address these limitations. For example, Sun et al. (2022) developed a vascularized human brain organoid model with improved functional maturation [300]. Another key constraint is limited incorporation. Most organoid systems lack resident or circulating immune cells, hindering comprehensive investigation of antiviral immunity and immune-mediated pathology [301]. Although co-culture strategies have improved immunological relevance, these systems remain simplified relative to native tissues. Donor-to-donor variability represents an additional challenge, particularly for adult stem cell-derived organoids. Genetic background, epigenetic state, and tissue origin influence viral susceptibility and host responses, complicating standardization. At the same time, this variability reflects clinically relevant heterogeneity and may be leveraged to investigate host-specific determinants of infection and therapeutic response.

Finally, technical limitations related to batch variability, protocol complexity, and reliance on extracellular matrices such as Matrigel continue to impede scalability and reproducibility [302,303,304]. Standardized culture conditions, synthetic matrices, and automation will be essential for broader clinical and translational deployment.

## 9. Conclusions

The rapid integration of organoid technology into virology research has provided an unprecedented, human-centric platform for unraveling the complexities of viral pathogenesis and accelerating therapeutic discovery. As demonstrated across numerous studies, these 3D models faithfully recapitulate critical disease hallmarks that conventional 2D cultures and non-human animal models often fail to capture.

The versatility of organoids is evident in two major fields. In neurovirology, brain organoids enabled direct visualization of ZIKV targeting neural progenitor cells, establishing a mechanistic link to microcephaly [68,305]. Incorporation of microglia further permitted modeling of DENV- and HIV-1-induced neuroinflammation [109,306]. In enteric virology, intestinal organoids overcame longstanding barriers to HuNoV cultivation, enabling analysis of FUT2-dependent host susceptibility [128,130,132]. During the COVID-19 pandemic, respiratory and intestinal organoids rapidly defined SARS-CoV-2 epithelial tropism and revealed altered infectivity profiles of emerging variants such as Omicron [160,169,307,308].

Looking forward, integration with microfluidic and organ-on-chip platforms is expected to enhance vascularization, inter-organ communication, and modeling of systemic infection. Parallel incorporation of adaptive immune components will deepen understanding of antiviral immunity and immune-mediated pathology. Together, these advances organize organoids as standardized preclinical platforms that can accelerate antiviral development, vaccine evaluation, and preparedness for future infectious disease threats. Such systems also enable systematic interrogation of host dysregulated pathways, including oxidative stress responses, as adjunct therapeutic targets [309,310].

## Figures and Tables

**Table 1:** Brain Organoids for Neurotropic Viruses. Summary of neurotropic virus studies using human brain organoids derived from pluripotent stem cells. The table highlights how organoid models have enabled the recapitulation of viral tropism, pathogenesis, and host–virus interactions relevant to the central nervous system (CNS). For each virus, the genome type, organoid model employed, principal neuropathological findings, and key studies are provided. Collectively, these findings demonstrate that brain organoids serve as physiologically relevant models to study viral replication, neurodevelopmental disruption, immune responses, and therapeutic interventions in human-relevant neural contexts.

Virus	Genome Type/ Family	Organoid System Used	Key Findings/Neuropathological Outcomes	Studies
Zika Virus (ZIKV)	+ssRNA/*Flaviviridae*	iPSC- or ESC-derived brain and forebrain organoids	Preferential infection of NPCs; apoptosis and impaired neurogenesis; organoid growth reduction (microcephaly-like phenotype); TLR3 involvement; neuroprotection by betulinic acid; oncolytic activity in GBM stem cells	[65,66,67,68,69,70,71]
Herpes Simplex Virus Type-1 (HSV-1)	dsDNA/*Orthoherpesviridae*	hiPSC- and hESC-derived brain organoids; hiNSC scaffold models	Acute and latent infection; disrupted neuronal differentiation and cortical layering; microglial activation; Aβ42 accumulation (AD-like pathology); impaired IFN signaling (ICP34.5-dependent); latency reduced by CRISPR targeting ICP0/ICP27	[74,76,77,78,79,80,81,123]
SARS-CoV-2	+ssRNA/*Coronaviridae*	Cortical, choroid plexus, CSF, and vascularized brain organoids	Infection of NPCs and cortical neurons; neuronal loss and reduced proliferation; high susceptibility of choroid plexus with CPE; spread via vascular/pericyte routes; ACE2-dependent and -independent entry (NRP1, TPCN1); variant-dependent neurotoxicity; antiviral sensitivity	[86,87,88,89,90,91,92,93,94,95,96,97]
Human Cytomegalovirus (HCMV)	dsDNA/*Orthoherpesviridae*	Brain, cerebral, cortical, and glioblastoma organoids	Severe cortical disorganization; reduced organoid size; necrosis and vacuolation; targeting of TBR2^+^ NPCs; transcriptional dysregulation (FEZF2, EMX1, FOXG1); mitochondrial dysfunction; EphA2-mediated entry in GBOs; AD-associated microglial signatures	[100,101,102,103,104,105,106,107,108]
Dengue Virus (DENV)	+ssRNA/*Flaviviridae*	iPSC-derived brain organoids with microglia	Robust neuroinflammatory response driven by microglia (IL-6, IL-1β, TNF-α, CCL2); lipid-raft–dependent infection; inhibition by hernandonine	[109,110]
Japanese Encephalitis Virus (JEV)	+ssRNA/*Flaviviridae*	hESC-derived cortical organoids	Developmental stage-dependent susceptibility; impaired IFN signaling in early organoids; activation of RIG-I/IFN-β/STAT1 in mature organoids; preferential infection of outer radial glia; microcephaly-like defects	[112]
Measles Virus (MeV)	−ssRNA/*Paramyxoviridae*	Brain organoids	Hyperfusogenic F-protein mutation (L454W) drives receptor-independent cell fusion and CNS spread; recapitulates measles inclusion body encephalitis	[115]

**Table 2:** Summary of enteric virus studies using intestinal organoids. Intestinal organoids, including human intestinal organoids (HIOs), human intestinal enteroids (HIEs), porcine intestinal organoids (PIOs), and organoids from other species, serve as physiologically relevant models for studying enteric viruses. These organoids allow detailed insights into viral tropism, replication kinetics, host–pathogen interactions, antiviral responses, and therapeutic interventions. Studies also leveraged organoids for mechanistic analysis, CRISPR-based identification of host factors, and preclinical antiviral testing.

Virus	Genome Type /Family	Organoid System Used	Key Findings/Neuropathological Outcomes	Studies
Human Norovirus (HuNoV)	Caliciviridae, +ssRNA	HIOs, HIEs	Sustained replication in enteroids; mature enterocyte tropism; FUT2-dependent susceptibility; platform for antiviral testing	[130,132,134,135,137]
Rotavirus (RV)	Reoviridae, dsRNA	HIOs	Productive infection with CPE; replication restricted by interferons; epithelial repair responses observed	[150,151,152,159]
SARS-CoV-2	Coronaviridae, +ssRNA	HIEs, HIOs, macrophage-augmented organoids	Enterocyte infection and GI tropism; ACE2/TMPRSS2-dependent entry; variant-specific infectivity; antiviral evaluation	[94,160,161,165,168,173]
Enterovirus (EV-A71, CVB2, PV3, EV-D68, EV7)	Picornaviridae, +ssRNA	HIOs, fetal colon organoids, porcine IOs	Efficient replication; goblet cell tropism; non-lytic viral release; identification of host factors	[177,178,181,182,183,185]
Transmissible Gastroenteritis Virus (TGEV)	Coronaviridae, +ssRNA	Porcine IOs (PIOs)	Robust replication; induction of antiviral and inflammatory responses; modulation of epithelial renewal	[188,189,190,191,192]
Porcine Epidemic Diarrhea Virus (PEDV)	Coronaviridae, +ssRNA	PIOs, PEMCs	Sustained replication; segment-specific susceptibility; host factors regulate entry and barrier integrity	[194,195,196,197,198,199]
Middle East Respiratory Syndrome (MERS-CoV)	Coronaviridae, +ssRNA	HIOs, bat-derived IOs	Productive intestinal infection; strong type III interferon response	[200,201]
Human Astrovirus (HAstV)	Astroviridae, +ssRNA	HIOs	FcRn identified as entry receptor; broad epithelial cell infection	[203,204]
Mammalian Reovirus (MRV)	Reoviridae, dsRNA	HIOs, PIOs	Type III interferon–mediated epithelial antiviral defense	[205,206]
Avian Influenza Virus (AIV) H9N2, H6N1	Orthomyxoviridae, −ssRNA	Mouse IOs, Chicken IOs	Apical infection recapitulates natural exposure; epithelial damage and ISG induction	[207,208]
Other enteric viruses (HCoV-OC43, BCoV, PDCoV, RCV-A1)	Coronaviridae /Caliciviridae	PIOs, bovine enteroids, chicken IOs, rabbit IOs	Host range and species-specific susceptibility assessed	[209,210,211,212]

**Table 3:** Summary of hepatotropic virus studies using liver organoids. Liver organoids (LOs), derived from adult stem cells (ASCs) or pluripotent stem cells (PSCs), serve as physiologically relevant models to study hepatotropic virus replication, host–pathogen interactions, antiviral immunity, and therapeutic interventions. These models replicate polarization-dependent entry, viral replication kinetics, immune responses, and disease-relevant pathology, and allow *ex vivo* testing of antiviral drugs and oncolytic virus efficacy.

Virus	Genome Type/Family	Organoid System Used	Key Findings/Neuropathological Outcomes	Representative Studies
Hepatitis B Virus (HBV)	Hepadnaviridae, dsDNA	PSC-derived LOs, patient-derived LOs, Intrahepatic cholangiocyte organoids (ICOs)	Productive infection with cccDNA formation; hepatic dysfunction and oncogenic signatures; platform for antiviral and host-targeted drug testing	[213,214,217,218,219,220,221,222]
Hepatitis C Virus (HCV)	Flaviviridae, +ssRNA	PSC- and ASC-derived LOs, multicellular LOs with Kupffer-like cells	Polarization-dependent viral entry; lipid metabolic dysregulation; enables study of adaptive immune interactions	[224,225,226,227,228]
Hepatitis E Virus (HEV)	Hepeviridae, +ssRNA	Fetal/adult LOs, polarized monolayers	Complete replication and viral shedding; interferon-mediated antiviral responses; drug screening feasible	[229]
Hepatitis D Virus (HDV)	Deltaviridae, −ssRNA satellite virus	Primary hepatocyte-like LOs	Immune activation via JAK/STAT signaling; reduction in viral burden	[223]
Dengue Virus (DENV)	Flaviviridae, +ssRNA	hPSC-derived LOs	Hepatocyte infection with mitochondrial injury; oxidative stress–linked pathology; antiviral compound screening	[243]
Oncolytic Viruses (OVs)	Adenoviridae, Herpesviridae, Caliciviridae	Human LOs, hepatobiliary organoids	Evaluation of oncolytic efficacy and immune activation; modulation of antiviral immunity alters therapeutic outcome	[230,231,232,233,234,235]
Non-canonical hepatotropic and liver-tropic viruses (DENV, SARS-CoV-2)	Flaviviridae /Coronaviridae, +ssRNA	hPSC-derived liver organoids; adult hepatocyte and cholangiocyte organoids	Productive hepatocyte and cholangiocyte infection; mitochondrial injury, oxidative stress, and inflammatory cytokine induction; IL-6–associated bystander responses; direct cytopathic effects contribute to virus-associated liver injury	[236,237,238]

**Table 4:** Summary of lung organoid models for the study of respiratory viruses. This table summarizes the different respiratory viruses studied using lung organoids, including airway organoids (AOs) and alveolar organoids (ALOs). For each virus, the virus family, the specific organoid model used, and key findings, including viral tropism, pathogenesis, immune responses, and antiviral testing, are highlighted. References are provided for further details on the methodologies and experimental outcomes. The table emphasizes how lung organoids recapitulate the cellular diversity of the respiratory tract and serve as platforms for mechanistic studies and preclinical antiviral evaluation.

Virus	Genome Type/Family	Organoid System Used	Key Findings/Neuropathological Outcomes	Representative Studies
SARS-CoV-2	Coronaviridae, +ssRNA	hESC- and ASC-derived airway/ alveolar organoids	Infection of ciliated and alveolar epithelial cells; strong inflammatory responses; epithelial damage recapitulating COVID-19 pathology; platform for antiviral evaluation	[248,249,250,251,252]
Influenza Virus (IAV)	Orthomyxoviridae, −ssRNA	Airway organoids, ALI	Strain-specific epithelial tropism and replication efficiency; airway damage and remodeling reflect virulence differences	[250,251,252,253]
Human Respiratory Syncytial Virus (HRSV)	Pneumoviridae, −ssRNA	Fetal and induced airway organoids, apical-out organoids	Maturity-dependent infection of airway epithelium; cytoskeletal disruption; enables neutralization and entry studies	[255,256,257,258]
Parainfluenza Virus (PIV)	Paramyxoviridae, −ssRNA	Airway organoids, bronchial/tracheal ALI cultures	Productive infection with preserved viral characteristics; supports antiviral testing and temperature-dependent replication studies	[261,262]

**Table 5:** Summary of kidney organoid models for viral infection studies. The table presents an overview of viruses studied in human kidney organoids (KOs), detailing the virus family, organoid type, and key findings on viral tropism, replication, cytopathic effects, immune response, and therapeutic interventions. The table highlights the use of kidney organoids in modeling proximal tubule infection, elucidating antiviral signaling pathways, and validating antiviral therapies. References provide additional experimental context and outcomes for each virus.

Virus	Genome Type/Family	Organoid System Used	Key Findings/Neuropathological Outcomes	Representative Studies
SARS-CoV-2	Coronaviridae, +ssRNA	iPSC-derived kidney organoids (KOs)	Infects proximal tubules via ACE2/TMPRSS2; productive replication, apoptosis, cell morphology changes; upregulates IFN pathways; therapeutic testing: spike binder peptides, soluble ACE2 1-618-ABD, remdesivir combination therapy	[271,272,273]
Mpox Virus (MPXV)	Poxviridae, dsDNA	iPSC-derived kidney organoids	Susceptible to infection; antiviral responsiveness demonstrated	[272,273,274]

## Data Availability

No new data were created or analyzed in this study. Data sharing is not applicable to this article.

## Abbreviations

2D: Two-Dimensional; 3D, Three-Dimensional; ACE2, Angiotensin-Converting Enzyme 2; AD, Alzheimer’s Disease; ALI, Air-Liquid Interface; AMPK, AMP-Activated Protein Kinase; ASC, Adult Stem Cell; AT2, Alveolar Type II Cell; AXL, AXL Receptor Tyrosine Kinase; BBD, Box–Behnken Design; BCA, Bicinchoninic Acid; BMP, Bone Morphogenetic Protein; CNS, Central Nervous System; CPE, Cytopathic Effect; CRISPR, Clustered Regularly Interspaced Short Palindromic Repeats; CSF, Cerebrospinal Fluid; DAB, 3,3′-Diaminobenzidine; DENV, Dengue Virus; dsDNA, Double-Stranded DNA; dsRNA, Double-Stranded RNA; EGFR, Epidermal Growth Factor Receptor; ESC, Embryonic Stem Cell; FXR, Farnesoid X Receptor; GBM, Glioblastoma Multiforme; GI, Gastrointestinal; GROMACS, GROningen MAchine for Chemical Simulations; HBGA, Histo-Blood Group Antigen; HBV, Hepatitis B Virus; HCMV, Human Cytomegalovirus; HCV, Hepatitis C Virus; HEK293T, Human Embryonic Kidney 293T Cells; HEV, Hepatitis E Virus; HIE, Human Intestinal Enteroid; HIO, Human Intestinal Organoid; HIV, Human Immunodeficiency Virus; HRSV, Human Respiratory Syncytial Virus; HSV-1, Herpes Simplex Virus Type 1; HuNoV, Human Norovirus; IAV, Influenza A Virus; IFN, Interferon; IgG, Immunoglobulin G; IHC, Immunohistochemistry; iPSC, Induced Pluripotent Stem Cell; ISC, Intestinal Stem Cell; ISG, Interferon-Stimulated Gene; IVT, *In vitro* Transcription; JAK, Janus Kinase; JEV, Japanese Encephalitis Virus; KO, Knockout; LACV, La Crosse Virus; LDLR, Low-Density Lipoprotein Receptor; LO, Liver Organoid; MAPK, Mitogen-Activated Protein Kinase; MAVS, Mitochondrial Antiviral-Signaling Protein; MD, Molecular Dynamics; MeV, Measles Virus; miRNA, MicroRNA; MPXV, Mpox Virus; MRV, Mammalian Reovirus; MSN, Mesoporous Silica Nanoparticle; NPC, Neural Progenitor Cell; NRP1, Neuropilin-1; PAMP, Pathogen-Associated Molecular Pattern; PBMC, Peripheral Blood Mononuclear Cell; PCR, Polymerase Chain Reaction; PEDV, Porcine Epidemic Diarrhea Virus; PEI, Polyethylenimine; PIO, Porcine Intestinal Organoid; PSC, Pluripotent Stem Cell; qRT-PCR, Quantitative Reverse Transcription Polymerase Chain Reaction; RNA-seq, RNA Sequencing; RV, Rotavirus; SARS-CoV-2, Severe Acute Respiratory Syndrome Coronavirus 2; scRNA-seq, Single-Cell RNA Sequencing; SFTS, Severe Fever with Thrombocytopenia Syndrome; SOCE, Store-Operated Calcium Entry; STAT, Signal Transducer and Activator of Transcription; TLR, Toll-Like Receptor; TMPRSS2, Transmembrane Serine Protease 2; uORF, Upstream Open Reading Frame; VLP, Virus-Like Particle; VOC, Variant of Concern; Wnt, Wingless/Integrated Signaling Pathway; ZIKV, Zika Virus; +ssRNA, Positive-Sense Single-Stranded RNA; −ssRNA, Negative-Sense Single-Stranded RNA.
